# Vagus nerve stimulation as a potential treatment for acute asthmatic bronchoconstriction: a systematic review

**DOI:** 10.3389/fphys.2025.1625871

**Published:** 2025-08-13

**Authors:** Elizabeth Di Flumeri, Francine M. Ducharme, Joël St-Pierre, Farbod Niazi, Nathan A. Shlobin, Simon Couillard, Jean-Paul Praud, Alexander G. Weil, Christian Iorio-Morin

**Affiliations:** ^1^ Department of Pediatrics, McGill University, Montreal, QC, Canada; ^2^ Departments of Pediatrics and of Social and Preventive Medicine, Sainte-Justine University Hospital, University of Montreal, Montreal, QC, Canada; ^3^ Division of Neurosurgery, Department of surgery, Université de Sherbrooke, Sherbrooke, QC, Canada; ^4^ Neurosurgery Service, Department of Surgery, Sainte-Justine University Hospital, University of Montreal, Montreal, QC, Canada; ^5^ Department of Neurological Surgery, Feinberg School of Medicine, Northwestern University, Chicago, IL, United States; ^6^ Respirology Service, Department of Medicine, University of Sherbrooke, Sherbrooke, QC, Canada; ^7^ Division of Pediatric Pulmonology, Department of Pediatrics, University of Sherbrooke, Sherbrooke, QC, Canada

**Keywords:** vagus nerve stimulation, neuromodulation, asthma, bronchoconstriction, respiratory function

## Abstract

**Objective:**

Vagus nerve stimulation (VNS) is a therapeutic option for diseases such as epilepsy and depression. Given that the smooth muscle of the bronchi is innervated by the vagus nerve, VNS could aid in treating pathologies of the respiratory system involving a bronchoconstrictive component. The aim of this review is to evaluate the literature on the potential for VNS to relieve airway bronchoconstriction in asthma.

**Methods:**

A systematic review of several databases (PubMed, Embase and Scopus) was conducted according to the PRISMA guidelines. Studies of individuals (humans and animals) with asthma symptoms were included if they reported respiratory function outcomes. Two authors independently reviewed all papers for selection, methodological assessment, and data extraction.

**Results:**

A total of 2072 articles were identified, of which 1,528 unique articles were screened for inclusion. 30 relevant articles underwent full-text review, and six articles (four in humans; two in animals) were included. One human article was subsequently added manually due to a new finding in an updated search. Due to incomplete data reporting, meta-analysis was not possible. In both animal studies, low-voltage VNS improved respiratory function following a histamine or acetylcholine challenge. In human studies (one case report, two prospective interventional cohort studies, one randomized controlled study), VNS, in addition to standard-of-care anti-asthmatic therapy, appears to temporarily improve respiratory function.

**Conclusion:**

Limited low-quality evidence suggests low-voltage VNS appears to reduce bronchoconstriction in both animal and human subjects. Given concerns about translatability, the absence of a control group in most studies, and the concomitant use of anti-asthmatic pharmacotherapy, it is not possible to draw conclusions about the true magnitude of VNS’s effect on respiratory function and acute asthma progression. Well-designed randomized controlled trials (RCT) are needed to further evaluate the effectiveness of VNS in treating acute asthmatic bronchoconstriction and to better understand its underlying therapeutic mechanisms.

## 1 Introduction

Asthma is a chronic respiratory disease affecting 11% of the Canadian population (Canada PHA of, 2018) and over 400 million people worldwide (To et al., 2012). Asthmatics have increased rates of work and school absenteeism, activity limitation and impaired quality of life (Nurmagambetov et al., 2018). Despite significant pharmacological advances over the last decades, asthma remains the third leading cause of hospitalization in children (Miner et al., 2012), increases all-cause mortality in children and young adults (Caffrey et al., 2020), and is responsible for over 250,000 preventable deaths worldwide annually (Enilari and Sinha, 2019). This obstructive lung disease imposes a significant socio-economic burden, particularly on medical resources. In 2013, the annual cost of asthma reached US$81.9 billion in the United States alone (Nurmagambetov et al., 2018).

Asthma is a heterogeneous disease and patients differ in their clinical presentation as a result of individual differences in the magnitude of various contributors to airway obstruction (inflammation, mucus, bronchospasm) as well as their genetically-determined response to triggers and pharmacological agents (Miner et al., 2012). The mainstay of asthma therapy rests primarily on pharmacological agents added in a consistent incremental sequence to target the key components of the disease: persistent airway inflammation with ongoing recruitment of inflammatory cells and associated edema, increased mucus production, and bronchial hyperresponsiveness (Global Initiative for Asthma - GINA, 2022; Hammad and Lambrecht, 2021). These include inhaled or systemic glucocorticoids (and/or anti-leukotrienes) to curtail inflammation, inhaled short and long-acting β2-adrenergic receptor agonists to relieve bronchoconstriction, as well as inhaled muscarinic antagonists. Despite their efficacy, chronic use of short- or long-acting β2-agonists may be associated with the development of tachyphylaxis in some patients (Doeing and Solway, 2013). Furthermore, the severe airway obstruction experienced by patients with poorly-controlled asthma or an acute asthma exacerbation, could interfere with inhaled pharmacological treatment as delivery to the target organs may be significantly limited, rendering management sub-optimal (Sepulveda et al., 2008).

Two anatomically and physiologically distinct parasympathetic pathways with opposing functions control airway tone (Canning et al., 2012). Cholinergic preganglionic neurons originating in the brainstem are responsible for the cholinergic drive provided to the airway ganglia. Cholinergic postganglionic neurons produce bronchoconstriction, whereas noncholinergic neurons induce bronchodilatation through the actions of nitric oxide and vasoactive intestinal peptide (Canning et al., 2012; Canning, 1985). Specifically, the vagus nerve innervates the smooth muscle of the bronchi, releasing acetylcholine and inducing bronchoconstriction upon activation, while the non-cholinergic parasympathetic fibers can exert the opposite effect by reducing bronchoconstriction. Thus, vagal parasympathetic innervation contributes to both the contraction and relaxation of bronchial smooth muscle (Canning, 1985). The appropriate balance between parasympathetic and sympathetic tone relies in part on vagal afferent signaling, which contributes to the regulation of lower airway smooth muscle tone and airway patency through physiological afferent-efferent reflexes (Canning, 1985). Given its role in the cholinergic peripheral release mechanism, and the clinical efficacy of anti-muscarinic agents in asthma and other respiratory diseases characterized by deleterious bronchoconstriction, the vagus nerve represents a potential therapeutic target for addressing the bronchoconstrictive component of asthma pathophysiology.

As with all nervous structures, vagus nerve function can be modulated through controlled electrical stimulation. Surgical vagus nerve stimulation (VNS) is approved for the treatment of epilepsy and depression and is routinely performed in many neurosurgical centers (Yap et al., 2020). Three other minimally or non-invasive VNS modalities (auricular, percutaneous, transcutaneous) exist and are mostly used in research settings. While VNS has been extensively studied for these approved indications and is currently being investigated for various systemic diseases influenced by vagus nerve activity, little data is available on its impact on respiratory function. One theoretical caveat lies in the apparent paradox of how stimulating the vagus nerve, a bronchoconstrictive pathway, could instead produce bronchodilatory effects.

The goals of this systematic review are to: (i) summarize the available data regarding the impact of VNS in relieving acute bronchoconstriction in animal and human subjects, (ii) identify intervention characteristics, namely, stimulation parameters and modality associated with successful relief of airway obstruction in humans, and (iii) ascertain the safety profile of this approach in humans. Our overarching goal is to elucidate whether VNS should be further explored as a promising approach to alleviate asthma in humans.

## 2 Methods

### 2.1 Study design and review protocol

This systematic review was conducted in accordance with the standards established by the Preferred Reporting Items for Systematic Reviews and Meta-Analysis (PRISMA) statement (Moher et al., 2009; Liberati et al., 2009). The study was not registered *a priori*; it was supported by existing local funds.

### 2.2 Data sources and search strategy

A comprehensive search for all relevant literature on VNS use was conducted on 10 July 2022, on three electronic databases: PubMed, Embase and Scopus using the search terms detailed in Supplementary Table S1. No restrictions on article type were applied. The search was last updated on 1 October 2023.

### 2.3 Study selection criteria

Studies were eligible for inclusion if they: (i) were a (pre-, quasi-, or true) experimental design, (ii) included adult humans or animal subjects, (iii) focused on asthma pathology or an element of asthma pathophysiology (e.g., bronchoconstriction), (iv) tested VNS as an intervention, and (v) reported respiratory function outcomes (e.g., change in forced expiratory volume in 1 s [FEV_1_], work of breathing [WOB], or pulmonary inflation pressure [Ppi] in mechanically-ventilated animals, for example,). Exclusion criteria included: (i) not original articles (e.g., review articles): or not full-text publications (e.g., abstracts), (ii) studies involving neuromodulation techniques other than VNS (e.g., deep brain stimulation), (iii) studies evaluating pharmacotherapy alone, and (iv) non-English language studies (Table 1).

**TABLE 1:** PICOS table for study selection criteria of the systematic review.

PICOS framework	Inclusion criteria	Exclusion criteria
Population	• Adult human subjects • Animal subjects • Study focusing on asthma pathology or an element of asthma pathophysiology (e.g., bronchoconstriction)	*No criteria*
Intervention	VNS	Neuromodulation other than VNS
Comparator	*No criteria*	*No criteria*
Outcomes	Reported respiratory outcomes	*No criteria*
Study design	Pre-, quasi-, and true experimental design	• Not original articles • Not full-text publications • Evaluating pharmacotherapy alone

VNS: vagus nerve stimulation.

### 2.4 Intervention

**FIGURE 1:**
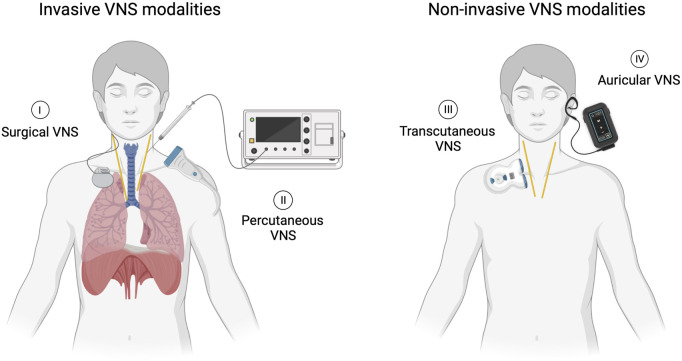
Four distinct VNS modalities. (I) Surgical VNS involving a permanently implanted lead and pulse generator. (II) Percutaneous VNS accomplished through direct stimulation of the nerve using an external stimulator connected to an electrode temporarily inserted under ultrasound guidance. (III) Transcutaneous VNS delivered by applying current on the skin overlying the nerve using an external stimulator. (IV) Auricular VNS stimulating vagal cutaneous afferents in the ear using a wearable surface electrode connected to an external stimulator.

The duration, amplitude of stimulation and type of VNS were documented. Specifically, the interventions were classified as one of four types of VNS (Figure 1). *Surgical VNS*, consisting of the surgical implantation of an electrode in the cervical portion of the vagal nerve connected to a subcutaneous pulse generator generally placed in the infraclavicular area. This system is permanent and provides long-term, continuous stimulation controlled wirelessly. *Percutaneous VNS*, referring to a temporary electrode inserted through the skin to the vagal nerve under ultrasound guidance. It is connected to an external stimulator and allows direct vagal nerve stimulation in an acute setting. *Transcutaneous VNS*, a non-invasive approach in which two electrodes are positioned over the skin on the trajectory of the vagal nerve. As current flows from one electrode to another, it stimulates the vagal nerve without the need for skin penetration. Lastly, *auricular VNS*, a non-invasive modality in which electrodes are positioned in the auricle of the ear, an area innervated by afferent sensory fibers from the vagus nerve. Its activation has been shown to generate cerebral activation patterns similar to surgical VNS (Yap et al., 2020). Invasive, minimally and non-invasive approaches are designed to target the main vagal pathway of the afferent and/or efferent fibers connecting the lungs with the brainstem and higher brain centers (Ouzzani et al., 2016). A comparator, sham VNS, where the device is applied without stimulation, can be utilized and studied, while not required for study selection in the review, it should be considered for assessing study design.

### 2.5 Data extraction and assessment

All references were imported into Rayyan, an online collaborative review tool (Ouzzani et al., 2016). Titles and abstracts of all imported articles were initially screened for relevance to the study topic. Articles that passed the initial screening were then evaluated in full text for final inclusion. At this stage, reference lists of all included articles were also reviewed to identify additional relevant studies that may have been missed in the initial search. All screening steps were independently performed by two authors (E.D.F. and F.N.). Any conflicts at any stage were resolved through discussion between the two authors or, if needed, through consultation with senior authors (A.G.W., C.I.M.).

Relevant data from the included studies was extracted and entered in a Microsoft Excel spreadsheet. The extracted variables included population type, number of subjects, patient age, and sex or animal species (as applicable), VNS modality, stimulation parameters, length of stimulation, reported adverse events and respiratory outcomes measures before and after VNS. When missing, the clinicaltrials.gov website was searched for potential additional results relative to registered trials.

### 2.6 Risk of bias assessment

The risk of bias was assessed by two authors and conflict was resolved by consensus. The Systematic Review Centre for Laboratory Animal Experimentation (SYRCLE) tool (Hooijmans et al., 2014) was used to evaluate the animal studies. The Institute of Health Economics (IHE) quality appraisal checklist for case studies (Institute of Health Economics, 2022) for both prospective interventional non-randomized human studies (Miner et al., 2012; Steyn et al., 2013) and the randomized placebo controlled trial (mehmed et al., 2017) and the Joanna Briggs Institute (JBI) critical appraisal tool (JBI Global, 2022) for the human case report (Sepulveda et al., 2008). The risk of publication bias was assessed using the Egger test.

## 3 Results

### 3.1 Study selection

**FIGURE 2:**
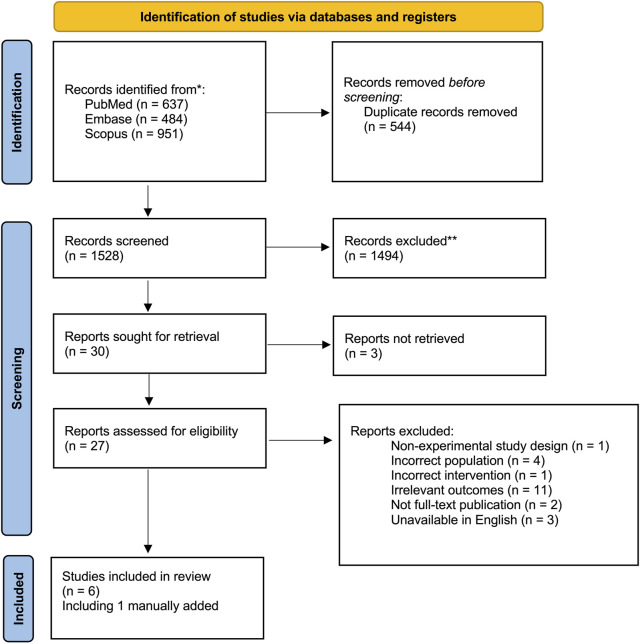
PRISMA flow diagram of manuscript selection.

In total, 2072 articles identified through the search strategy, after removal of duplicates, 1,528 citations were screened for inclusion, 30 articles were reviewed in full text, of which five were included for analysis in this review. One article from the grey literature was added manually as it was found accidentally when co-authors were reviewing the manuscript and was not generated as a result in the initial search as it was not indexed in the search engines used (Figure 2).

### 3.2 Study and participant characteristics

Of the six studies, two were animal randomized controlled trials (RCT) conducted in guinea pigs with/without swine and four were human studies (one case report, two non-randomized interventional studies without a control group and one randomized placebo-controlled study) (Table 2).

**TABLE 2:** Animal and human study characteristics.

Author and year	Study location	Study design	Number of subjects (N)	Animal species	VNS modality
Animal studies					
Hoffman et al. (2009)	United States	Randomized controlled trial	Guinea pigs: N = 16 (Histamine challenge) Swine: N = 3 (Histamine challenge)	Male Hartley guinea pigs and Yorkshire domestic cross swine	Invasive surgical
Hoffman et al. (2012)	United States	Randomized controlled trial	Guinea pigs: N = 26 (Histamine challenge) Guinea pigs: N = 4 (Acetylcholine challenge)	Male Hartley guinea pigs	Invasive surgical

Author and year	Study location	Study design	Number of participants (N)	Participant characteristics	VNS modality
Human studies					
Sepulveda et al. (2008)	United States	Case report	N = 1	34 years old Male 100%	Invasive percutaneous
Miner et al. (2012)	United States	Prospective interventional non-randomized	N = 24	18–65 years old (median 41) Male 36%, Female 64%	Invasive percutaneous
Steyn et al. (2013)	South Africa	Prospective interventional non-randomized	N = 4	18–70 years old (median NR) Sex (%) NR	Non-invasive transcutaneous
Mehmed et al. (2017)	Egypt	Randomized controlled trial	N = 31	21–70 years old (median NR) Sex (%) NR	Non-invasive transcutaneous

NR: not reported.

### 3.3 Evaluation of biases

For both included animal studies, the risk of bias per the SYRCLE tool (Hooijmans et al., 2014) was low or unclear because insufficient details were provided (Supplementary Table S2). For the single human case report (Sepulveda et al., 2008), the risk of bias was low when using the JBI tool (JBI Global, 2022) (Supplementary Table S3). For the three remaining human studies (Miner et al., 2012; Steyn et al., 2013; mehmed et al., 2017), the risk of bias was “variable” depending on the type of bias assessed when using the IHE tool for case studies (Supplementary Table S4). Of note, the study by Mehmed et al. (mehmed et al., 2017) has several methodological limitations, but was included by consensus as its findings are directly related to the research question in this review.

### 3.4 Animal studies

Both animal studies assessed the ability of a surgical VNS procedure to attenuate histamine-induced (Hoffmann et al., 2009; Hoffmann et al., 2012) and acetylcholine-induced (Hoffmann et al., 2012) bronchoconstriction in guinea pigs (n = 16) and swine (n = 3) or guinea pigs alone (n = 34) during volume-controlled endotracheal ventilation. Both used similar voltage ranging from either 0.4–1.5 V (Hoffmann et al., 2009) to 0.5–2 V (Hoffmann et al., 2012). In both studies, succinylcholine injections were administered to induce chest wall muscle paralysis prior to provoking bronchoconstriction in order to eliminate contributions from possible chest wall contractions on airway measurements. Histamine and acetylcholine doses were chosen to elicit a 2–6 cm H2O increase in Ppi. In Hoffmann et al. (2009), electrical stimulation begun 3 s prior to the histamine challenge to account for histamine’s rapid onset time. The length of stimulation was not explicitly reported in the first study.

A second follow-up study by the same lead author was conducted in 2012 in attempts to elucidate the mechanisms by which low-voltage VNS reduces bronchoconstriction. Here, VNS was applied starting 20 s prior to the histamine challenge and continued for 10 s past the peak response. A histamine challenge was tested in 26 guinea pigs, whereas bronchoconstriction was elicited with acetylcholine in six guinea pigs. In a small subset of guinea pigs (n = 4), VNS was applied for a total of 30 s and discontinued 10 s prior to the histamine challenge. This was compared to a group of animals who had the VNS on for a total of 20 s before as well as throughout the histamine challenge. The main outcome in both studies was the peak change in pulmonary inflation pressure (Ppi) calculated as the difference between baseline and maximum response following a histamine challenge, with 2–6 cm H_2_O/L/s increase in Ppi being *a priori* identified as the maximal value targeted to avoid fatigue or adaptation. In Hoffmann et al. (2012), the ability of low-voltage VNS to attenuate histamine-induced increases in Ppi was studied by administering pharmacological inhibitors as well as cephalic and caudal vagus nerve ligation. Bronchodilators were not administered in either study.

In both animal species, the electrical stimulation significantly reduced histamine-induced bronchoconstriction measured by an average decrease in Ppi of about 1 cm H_2_O/L/s (Table 3). The latter is approximated as the authors did not report absolute change from baseline but only the group mean pre and post bronchoconstriction.

**TABLE 3:** Pulmonary inhalation pressure before and after low-voltage vagus nerve stimulation in guinea pig and swine models.

Authors and year	Stimulation parameters	Bronchoconstriction-inducing challenge	Timing of stimulation	Pre-VNS peak increase in Ppi	Post-VNS peak increase in Ppi	Change from baseline (p value)
Hoffman et al. (2009)	Frequency (Hz): 25 Pulse duration (ms): 0.2 Pulse amplitude (V): 0.4–1.5	Histamine challenge A) Guinea pig (*N* = 16) B) Swine (*N* = 3)	3s prior to histamine challenge	A) Guinea pig: 3.4 ± 0.4 cm H_2_O B) Swine: 3.3 ± 0.3 cm H_2_O	A) Guinea pig: 2.1 ± 0.2 cm H_2_O B) Swine: 2.4 ± 0.2 cm H_2_O	A) p < 0.001 B) p < 0.05
Hoffman et al. (2012)	Frequency (Hz): 25 Pulse duration (ms): 0.2 Pulse amplitude (V): 0.5–2.0	A) Histamine challenge (N = 26) B) Acetylcholine challenge (N = 6)	20s prior to histamine challenge to 10s after peak response	A) Guinea pig: 4.4 ± 0.3 cm H2O B) Guinea pig: 4.8 ± 0.9 cm H2O	A) Guinea pig: 3.2 ± 0.2 cm H2O B) Guinea pig: 3.1 ± 0.6 cm H2O	A) p < 0.01 B) p < 0.05

Hz: hertz, ms: millisecond, Ppi: pulmonary inflation pressure measured under constant-volume mechanical ventilation during a histamine challenge, V: volts.

### 3.5 Human studies

All four human studies report adults who presented to the emergency department (ED) with an acute asthma exacerbation. Only the RCT had a placebo-controlled group (mehmed et al., 2017). All studies provided standard-of-care anti-asthmatic medications concomitantly with VNS.

In all four human studies, FEV_1_ was reported at various time points after stimulation in various ways (mean group absolute values, mean/median % predicted value), with only one study (Miner et al., 2012) reporting the median within-patient change from baseline with a 95% CI (Table 4). In asthmatic adults, a change from baseline in FEV_1_ of at least 12% and at least 200 mL is considered clinically important (Global Initiative for Asthma - GINA, 2022). According to the American Thoracic Society (Jones et al., 2014), the validated minimal clinically important difference (MCID) for FEV_1_ is an absolute change of 100 mL (Donohue, 2005) or a relative change of 5%–10% (Cazzola et al., 2008) from baseline. In this review, three (Miner et al., 2012; Steyn et al., 2013; mehmed et al., 2017) of four human studies reported the % change in FEV_1_ after VNS in each group; 2 studies (Miner et al., 2012; Steyn et al., 2013) reported the number of individuals meeting a mean change from baseline of at least 12% from baseline; and only one 1 study (mehmed et al., 2017) had a control group. Due to incomplete data reporting, a meta-analysis was not possible, preventing the conduction of the Egger test, thus the results are hereafter reported narratively.

**TABLE 4:** Mean within-patient change from baseline post end of VNS in human studies.

Authors and year	Sepulveda et al. (2008)	Miner et al. (2012)	Steyn et al. (2013)	Mehmed et al. (2017)
Stimulations parameters	Frequency (Hz): 25 Hz Pulse duration (ms): 0.2 Pulse amplitude (V): ↑ until 7.5 Maintained at 7	Frequency (Hz): 25 Hz Pulse duration (ms): 0.2 Pulse amplitude (V): 1–12 (Median 4.4)	NR	Pulse amplitude (V): 9 (Other parameters NR)
Length of stimulation	180 min	60 min	2 nVNS treatments of 60 s each, 30 min apart	20 min
Baseline FEV_1_ (absolute and %predicted)	Absolute: 2.18 L Predicted: 69%	Absolute: NR Predicted: 34.1%	Absolute: NR Predicted: NR	Absolute: NR Predicted: 49.6%
Absolute change in %predicted FEV_1_ (95% CI)	NR	(Median change) Since beginning of stimulation 15 min: 15.80% (9.3%–22.4%) 30 min: 23.70% (8.1%–36.5%) 60 min: 27.50% (11.3%–43.5%) Post end of stimulation 30 min: 40.4% (21.4%–59.4%)	(Mean change) 2 min: 50% 15 min: 38.80% 30 min: 59.94% 60 min: 70.91% 90 min: 72.90%	(Mean change) 10.19% (timepoint: NR)
Absolute FEV_1_ (L)	Since beginning of stimulation 15 min: 3.18 L Post end of stimulation 30 min: 3.38 L 60 min: 2.84 L	NR	NR	NR
% change in WOB (95% CI)	NR	(Median change) Since beginning of stimulation 15 min: 53.9% (33.7%–73.9%) 30 min: 69.7% (56.4%–81.8%) 60 min: 81% (68.5%–93.5%) Post end of stimulation 30 min: 85.4% (67.4%–100%)	NR	NR
Dyspnea score	NR	NR	Baseline VAS for dyspnea: 7.65 2 min: 3.08 15 min: 3.70 30 min: 2.73 60 min: 1.85 90 min: 1.28	NR

N.B. P-values were not all reported in the above studies and 95% CI, were not always available.

FEV1: forced expiratory volume in 1 s; VAS: visual analog scale; WOB: work of breathing (maximum: 9; minimum: 0); SD: standard deviation; NR: not reported.

In 2008, Sepulveda et al. (2008) conducted the first published human case study to evaluate the potential clinical utility of a percutaneous VNS procedure in the treatment of acute asthmatic bronchoconstriction. The study presents a 34-year-old man with a 4-year history of severe asthma who presented to the ED during an acute exacerbation unresponsive to self-treatment with inhaled beta-adrenergic and steroid therapy. Over a 9-h period in the ED, he was administered nebulised albuterol (2.5 mg), oral prednisone (60 mg) and oral azithromycin (500 mg). After 5 h of treatment, a spirometry test revealed an FEV_1_ at 69% of the predicted value. After 8 h without clinical or spirometry (FEV_1_ 70% of predicted) improvement, the patient underwent ultrasound-guided placement of a percutaneous electrode. Voltage amplitude was gradually increased until the patient experienced a mild muscle twitch at 7.5 V; it was then lowered and maintained at 7 V throughout VNS, which lasted 180 min. Fifteen minutes following VNS initiation, FEV_1_ increased to 85% of predicted, which persisted during the 3-h stimulation window. Improvement in respiratory function was short-lived, as the effect did not persist longer than 30 min after cessation of the stimulation (Table 4).

Miner et al. (2012) carried out a prospective multi-center study in 25 moderate-to-severe asthmatic patients presenting to the ED with a FEV_1_ ranging from 25% to 75% of the predicted value after 1 hour of standard pharmacological therapy. Upon admission to the ED and throughout the study, participants received repeated doses of nebulized albuterol (5 mg) and ipratropium bromide (0.5 mg), oral corticosteroids (60 mg), and magnesium sulfate (2 g). Percutaneous VNS was continuously administered for 60 min with a mean peak stimulation of 4.4 V. The voltage amplitude was gradually increased until patients reported improvement in breathing, a muscle twitch or slight discomfort or until a maximum of 12 V was achieved. The mean baseline FEV_1_ was 34.1% of predicted value. Fifteen, 30, and 60 min after VNS was initiated, the median increase from baseline in FEV_1_ was 15.8% (n = 24), 23.7% (n = 22), and 27.5% (n = 21), reaching a median % predicted FEV_1_ of 49.9%, 57.8%, and 61.6%, respectively. Thirty minutes following VNS cessation, FEV_1_ improvement had reached a median of 40.4% (n = 20) from the pre-stimulation baseline with a median % predicted FEV_1_ of 74.5% (Table 4). In this study, treatment responders were *a priori* defined as having reached a 12% or greater increase from baseline in FEV_1_. The proportion of treatment responders was 54.2% at 15 min (n = 24), 77.3% at 30 min (n = 22), 81.0% at 60 min (n = 21), with 80.0% remaining responders 30 min (n = 20) after the end of stimulation. Improvement in perceived work of breathing (WOB), was assessed by a visual analogue scale, ranging from “normal breathing” to “extreme difficulty breathing”, in 16 of 25 patients. At 15, 30, and 60 min after initiation of stimulation, the WOB decreased by a median of 53.9% (n = 16), 69.7% (n = 15), and 81% (n = 14), respectively. A median 85.4% (n = 14) decrease from baseline in the WOB was observed 30 min after ending the stimulation. Although a clinically meaningful change has not yet been established for this outcome, treatment responders were *a priori* defined by authors as having attained at least a decrease 50% from baseline in WOB. At 15, 30 and 60 min of stimulation, 50% (n = 16), 80.0% (n = 15), and 85.7% (n = 14) of patients were classified as responders respectively.

Steyn et al. (2013) conducted a prospective multicenter study in which they intended to enroll 25 participants, but only four adult patients were eligible. Patients received two transcutaneous VNS treatments lasting 60 s each and separated by a 30-min interval, during which they continued to receive anti-asthmatic medications. A 12% improvement in FEV_1_, a 1.5 point or more improvement in the dyspnea score, and the absence of serious AEs were considered indicative of success. Outcomes were assessed at 15, 30, 60, and 90 min post-stimulation. The mean relative change from baseline in FEV_1_ was 38.8% at 15 min, 59.94% at 30 min, 70.91% at 60 min and 72.90% at 90 min post-stimulation. The mean dyspnea score (minimum: 0; maximum: 10), decreased significantly from 7.65 to 1.28 after stimulation (Table 4). Of four participants, 3 (75%) were classified as responders based on fulfillment of all 3 defined criteria above. Other *a priori* stated secondary outcomes included the time to discharge from the ED and the number of participants requiring additional anti-asthmatic medications that were not reported in the publication. Partially updated but unpublished results on *Clinicaltrials.gov* indicate an average time to discharge from the ED after stimulation of 193 min in six participants and all patients requiring anti-asthmatic medications post-stimulation (Steyn et al., 2013).

Finally, Mehmed et al. (2017) carried out a randomized placebo-controlled study in which 31 patients with bronchial asthma were recruited from the ED. Patient eligibility criteria included a confirmed asthma diagnosis, use of short-acting beta 2 agonist nebulizer during acute exacerbation, and no prior use of VNS. No mention was made of requirements regarding baseline severity, duration and intensity of anti-asthmatic ED treatment, or definition of insufficient response prior to enrolment. Participants were randomly assigned to one of 2 groups: 21 patients received 20 min of non-invasive transcutaneous VNS through 4 bilateral cervical electrodes (VNS group) while 10 patients underwent placement of the electrodes but did not receive stimulation (sham group). Stimulation was provided at 9V. Primary outcomes included pre- and post-stimulation pulmonary function test parameters: functional vital capacity (FVC), FEV_1_, peak expiratory flow (PEF) and forced expiratory flow at 25%–75% of FVC (FEF 25%–75%). However, the authors do not report the time points at which these parameters are measured. A significant difference in the paired t-test between pre and post treatment in percent of predicted FVC, FEV_1_, PEF, and FEF 25%–75% was reported in the VNS group, with increases of 10.76%, 10.19%, 10.43% and 10.40%, respectively, while no significant differences were reported in the sham group (increase of 0.4%, 1.7%, 5.5% and decline of 6.8%, respectively). The percentage of relative change between both groups was statistically significant for FEV_1_, PEF and FEF 25%–75%, but not significant for FVC.

### 3.6 Adverse events (AEs)

In all human studies, few AEs were reported during or after percutaneous or transcutaneous VNS. However, studies were not consistent or standardized in their reporting of AEs. In fact, during the VNS placement procedure, Miner et al. (2012) reported diaphoresis (n = 1), minor bleeding (n = 1) and a hematoma at the insertion site (n = 1), which resolved at the 7-day follow-up (Table 5). Vital signs (heart rate, mean arterial pressure, oxygen saturation and respiratory rate) were monitored and no significant changes were noted (Miner et al., 2012). Of the four participants tested, Steyn et al. (2013) assessed the presence of AEs throughout stimulation, at 7 days, and 30 days post-procedure. AEs reported included chest tightness (n = 1) and a respiratory tract infection (n = 1); the latter considered unrelated to the device or procedure. Miner et al. (2012)) reported cough (n = 1, moderate), temporary mild swallowing difficulty (n = 2), temporary mild hoarseness or voice change (n = 2) and muscle twitching (n = 14) during stimulation, rated as mild in 8, moderate in 5, and severe in one, participants: all reported AEs resolved after decreasing the stimulation intensity and did not persist during treatment (Table 5). Insertion of the VNS electrode and subsequent stimulation were uneventful and at the 7-day follow-up, no side effects were reported in the Sepulveda case report (Sepulveda et al., 2008). Although Mehmed et al. (2017) trial includes safety endpoints in its methods (serious and treatment-emergent AEs), these are not reported in the manuscript or on *clinicaltrials.gov* website.

**TABLE 5:** Reported adverse events across human studies.

Author and year	Sepulveda et al. (2008)	Miner et al. (2012)	Steyn et al. (2013)	Mehmed et al. (2017)
VNS modality	Percutaneous VNS	Percutaneous VNS	Transcutaneous VNS	Transcutaneous VNS
Events during device placement, n/N (%)				
Diaphoresis	NR	1/24 (4.2%)	NR	NR
Minor bleeding	NR	1/24 (4.2%)	NR	NR
Hematoma at insertion site	NR	1/24 (4.2%)	NR	NR
Events during the procedure, n/N (%)				
Chest tightness	NR	NR	1/4 (25%)	NR
Respiratory tract infection	NR	NR	1/4 (25%)	NR
Cough	NR	1/24 (4.2%)	NR	NR
Temporary hoarseness or voice change	NR	2/24 (8.4%)	NR	NR
Temporary swallowing difficulty	NR	2/24 (8.4%)	NR	NR
Muscle twitching	NR	14/24 (58.3%)	NR	NR

n: Number of events; N: total participants; NR: not reported.

## 4 Discussion

In all included studies, low-voltage VNS appears to be associated with a short-term improvement in respiratory function, characterized by a rapid response that is sustained throughout stimulation. However, the absence of a control group in all, but one human study, the concomitant use of standard-of-care pharmacotherapy and the lack of standardization in species, stimulation parameters including modality (surgical technique in animals vs percutaneous and transcutaneous approaches in humans), voltage, duration of stimulation and implementation relative to a bronchoconstriction inducing challenge, should prompt readers to interpret reported findings with caution as it is unknown if, and how much VNS independently contributed to the observed effect on respiratory function improvement. Although all these VNS modalities aim to target the same neurologic structure and seek reproducible effects, various aspects must be considered in the purpose of the application and evaluation, as they do not all produce equivalent specific electrophysiological influences on vagal activity. Surgical and percutaneous VNS aim to directly stimulate the vagus nerve, potentially modulating parasympathetic tone while activating afferent pathways that may increase sympathetic activity in the airways (Canning, 1985). Transcutaneous cervical and auricular VNS share more similar goal, mostly activating afferent vagal fibers to promote bronchodilation (Canning, 1985). The mere distinction in VNS modalities studied significantly limits generalization as the pathophysiological mechanisms and the side effect profile differ significantly from one modality to another. Each VNS modality should likely be considered as distinct interventions and studied separately to better comprehend their effects on respiratory function.

Persistence in FEV_1_ improvement was not assessed longer than seven or 30-day follow-ups, preventing assessment of the long-term effects of VNS on airway function, though, when assessed, the duration of effect was generally sustained for 30 min (Miner et al., 2012). However, Hoffman et al., 2012s evaluation of Ppi after discontinuation of low-voltage VNS when comparing animals in which VNS was applied for 30 s then discontinued 10 s prior to the histamine challenge with those who had the VNS left on throughout the histamine challenge, showed that low-voltage VNS remained effective at attenuating bronchoconstriction even when stimulation was discontinued. This prolonged response is consistent with the physiological mechanisms supporting the iNANC nervous system, which has a slower response and longer duration (Canning, 1985; Hoffmann et al., 2012). Though long-term respiratory outcomes were not studied in humans, the hypothetical pathophysiological response demonstrated in the guinea pig model may offer some background in beginning to understand whether, if any, the effects of low-voltage VNS on smooth muscle tone, can be sustained in time. Despite methodological pitfalls, the group difference in the magnitude of improvement in objective lung function tests reported by Mehmed et al. (2017) provides a hypothetical framework for assessing this intervention.

### 4.1 Proposed responsible mechanisms

**FIGURE 3:**
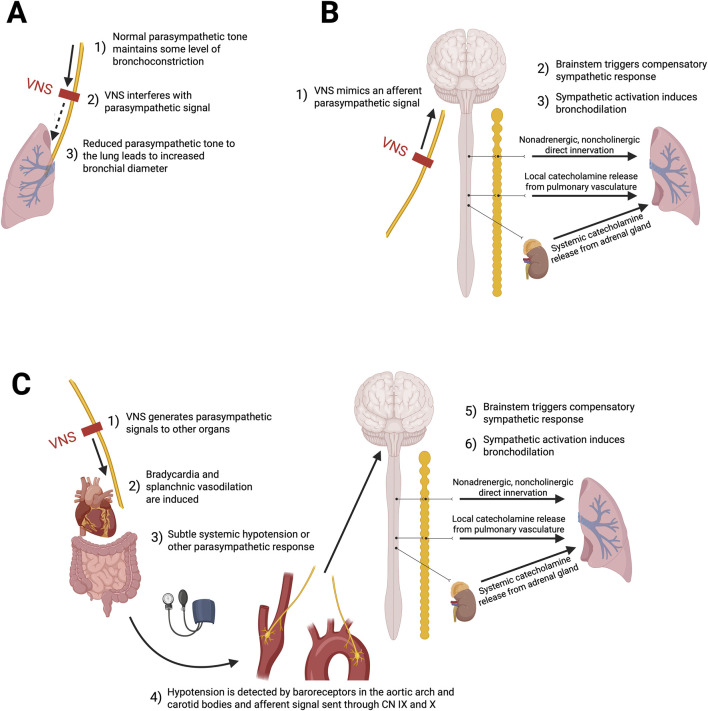
Putative hypothetical mechanisms of action through which low-voltage VNS might induce bronchodilation. **(A)** Inhibition of parasympathetic tone: VNS may suppress cholinergic parasympathetic activity, decreasing bronchoconstriction. **(B)** Reflex sympathetic activation through synthetic parasympathetic afferent: Afferent vagal signals may reach the brainstem and trigger reflex sympathetic responses that promote bronchodilation. **(C)** Reflex sympathetic activation through systemic parasympathetic response: Efferent vagus nerve stimulation may induce systemic parasympathetic effects, such as hypotension, leading to baroreceptor-mediated afferent signaling and a compensatory sympathetic response that favors bronchodilation.

The mechanisms through which VNS induces bronchodilation are likely multifactorial, but remain unclear. Leading hypothetical pathways are summarized in Figure 3. Activation of parasympathetic nerves, through the release of acetylcholine, evokes bronchoconstriction and mucous production in most species (Canning et al., 2012; Canning, 1985). As such, VNS might suppress, or interfere with, the parasympathetic signals descending on the vagal nerve towards the lungs, potentially leading to a desensitization of bronchial hyperreactivity and inhibition of bronchoconstriction. Under eupneic conditions, parasympathetic and sympathetic systems exert tonic control over the airways, so VNS may modulate this fine-tuned autonomic imbalance in order to promote bronchodilation (Canning, 1985) (Figure 3A). An alternative hypothesis may be that VNS generates a reflex increase in sympathetic tone. This could occur either by sending direct afferent parasympathetic signals to the brainstem through the vagus nerve (Figure 3B), or by generating a parasympathetic response (like a subtle hypotension) that would be compensated by sympathetic activation (Miner et al., 2012; Yuan and Silberstein, 2016) (Figure 3C). Postganglionic sympathetic nerves could then induce relaxation of airway smooth muscle. Given human airway smooth muscle typically lacks direct bronchodilatory sympathetic innervation could then occur through 3 potential mechanisms: 1) Systemic catecholamines secreted by the adrenal medulla directly binding to beta-adrenergic receptors on bronchial smooth muscle; 2) local release of epinephrine from the pulmonary vasculature (which is sympathetically innervated); or 3) via a distinct inhibitory nonadrenergic noncholinergic (iNANC) nervous system that remains to be fully characterized (Miner et al., 2012; Canning, 1985; Hoffmann et al., 2012). Hoffmann et al. (2012) study showed that the primary mechanism by which low-voltage VNS attenuates histamine-induced bronchoconstriction is through an afferent nerve pathway resulting in efferent stimulation of chromaffin cells causing catecholamine release. These catecholamines, specifically epinephrine, lead to smooth muscle airway relaxation through binding on β2-adrenergic receptors (Hoffmann et al., 2012). In fact, iNANC nerves have been shown to modulate tracheal and smooth muscle airway tone through the release of nitric oxide (Hoffmann et al., 2012). Even when a nitric oxide synthase inhibitor was administered, low-voltage VNS remained capable of attenuating bronchoconstriction (Hoffman et al., 2012) (Hoffmann et al., 2012). The work of Canning et al. (Canning, 1985) suggests that nonadrenergic noncholinergic neurotransmitters mediate the bronchial relaxation of the human airway smooth muscle. Additionally, vagus nerve activation of the cholinergic anti-inflammatory pathway has been studied as a potential neuroimmune modulator of pro-inflammatory cells and cytokines, and may represent a speculative beneficial mechanism in regulating asthma’s inflammatory pathophysiological components (Hammad and Lambrecht, 2021; Fang et al., 2023). More studies will be required to further define these mechanisms of action in humans.

### 4.2 Stimulation parameters and intervention characteristics

In animals, similar stimulation parameters were chosen to represent the lower spectrum of what is frequently used when applying VNS for the treatment of refractory epilepsy and depression in humans. This level of stimulation was also deemed safe as it was shown not to cause physiological damage to the vagus nerve (Hoffmann et al., 2009). In humans, stimulation parameters need to be further standardized to better comprehend both the required length of stimulation to produce an effect, if any, and the duration of the effect on airway tone and respiratory function. The lack of sustained evaluation of the impact on lung function beyond 60 min following the end of VNS also precludes any conclusion on the duration of sustained effect.

The parameters, specifically frequency and intensity, of VNS are critical in determining its physiological effects, as the vagus nerve contains both afferent fibers (A-, B-, and C-fibers) and efferent parasympathetic fibers. Low-voltage stimulation tends to preferentially activate afferent fibers, while high-voltage stimulation recruits both afferent and efferent fibers, potentially inducing more systemic parasympathetic effects such as bradycardia and bronchoconstriction (Capilupi et al., 2020; Li et al., 2024). This differential recruitment is probably due to the varying activation thresholds of fiber types. A- and B-fibers, being larger in diameter, have lower thresholds, whereas C-fibers, which are smaller and unmyelinated, require higher voltage stimulation (McAllen et al., 2018). In line with the proposed mechanism, activation of A- and B-afferent fibers may engage the reflex increase in sympathetic tone, whereas stimulation of C-fibers is more likely to trigger visceral efferent vagal responses (Bucksot et al., 2020; Jayaprakash et al., 2023; Castoro et al., 2011). Even though the trials reviewed employed different intervention modalities, they are all grounded in the same proposed mechanisms, where the stimulation of afferent vagal fibers would trigger a therapeutically desirable sympathetic reflex pathway that results in bronchodilation (Canning, 1985). While the modalities may differ in neurological selectivity and specificity, and magnitude of therapeutic response, they are thought to rely on a common mechanistic rationale (Miner et al., 2012; Yuan and Silberstein, 2016). Low-voltage stimulation appears more beneficial than high-voltage stimulation for achieving the desired bronchodilatory effect, but the precise mechanisms underlying these responses remain unclear (Hoffmann et al., 2009). Further studies are needed to elucidate how different stimulation parameters may differentially modulate the various fiber types of the vagus nerve.

### 4.3 Safety considerations

All AEs reported were temporary and did not require any additional intervention. During device placement, no instances of nerve damage were reported^4^. At the onset of stimulation, muscle twitching was reported in few patients but was used as a reference point to downward adjust voltage (Miner et al., 2012; Sepulveda et al., 2008). Cardiorespiratory vitals were monitored throughout stimulation and remained stable (Miner et al., 2012; Sepulveda et al., 2008). Importantly, in the small number of human subjects studied, no AEs were reported seven or 30 days later (Miner et al., 2012). These findings are comparable to those seen with the use of VNS in refractory epilepsy (Nemeroff et al., 2006). Despite the paucity of data and great heterogeneity amongst studies, percutaneous and transcutaneous VNS approaches in humans were reported to be safe. With the preliminary data reported above, it may be of interest for future clinical trials to focus on the use of transcutaneous VNS, as it poses a lesser risk of damaging the vagus nerve and its surrounding vessels. Further, the success of the percutaneous procedure is dependent on the experience of the user in the placement of both central and venous lines. This is supported by the safety data reported on the clinicaltrial.gov website for an unpublished trial (NCT01532817) entitled *Relief of Acute Bronchoconstriction/Asthma Using the Non-Invasive AlphaCore Device,* conducted in 30 humans receiving a single 90 s stimulation to the right vagus nerve (Electr oCore INC, 2018); this study was excluded from our review due to absence of published efficacy outcomes, both in the medical literature or online. In this study, minor AEs such as local neck pain, sensation of muscle tightness from right ear to arm or upper body, upper body jitteriness, hoarseness and viral respiratory syndrome, occurred in 30% of participants with no serious or delayed (at a 9-week follow-up) AEs reported.

### 4.4 Limitations and future work

Despite low risk of bias in animal studies and low-to-moderate risk of bias among included human studies, the level of evidence supporting the use of VNS to treat bronchoconstriction remains low. The reported animal studies (Hoffmann et al., 2009; Hoffmann et al., 2012) provide evidence from fundamental *in vivo* models on the effects of surgical VNS in reducing histamine and acetylcholine-induced bronchoconstriction. Although histamine is one of the most studied inflammatory mediators with the H1 receptor’s activity on the vagus nerve’s ability to trigger smooth muscle contraction, the histamine challenge used to induce bronchoconstriction does not fully recreate the complex pathophysiology of asthma, thereby limiting the translatability of these findings in asthmatic patients (Hoffmann et al., 2009). The acetylcholine-induced challenge in Hoffmann et al. (2012) is likely a better reflection that the predominant mechanism in bronchoconstriction is through activation of parasympathetic nerves causing the release of acetylcholine.

Although the results presented show promise for future work, the paucity of human trials, small sample sizes, largely heterogeneous stimulation parameters and protocols and the pre-experimental design without control groups in most studies warrant caution. Multiple biases must be accounted for when interpreting the data. Selective reporting, and consequently, publication biases inherent to the analysis of a limited number of clinically relevant published studies must be accounted for. Most importantly, the concomitant use of standard-of-care medications makes it difficult to isolate the specific effect of VNS on respiratory function. The absence of a placebo arm alongside standard-of-care treatment further limits the ability to assess the true therapeutic impact, particularly in asthma, where the placebo response (improvements observed in the placebo arm) can be substantial (Wechsler et al., 2011). This is driven by multiple factors, including the Hawthorne effect, in which increased adherence can significantly influence asthma outcomes and bias the assessment of the investigated treatment effect (Evans et al., 2021). Consequently, Mehmed et al. (2017) proposes a more robust framework for designing future trials aimed at evaluating improvements in respiratory function and symptom management. Fast-acting inhaled beta-agonists have an onset of action time ranging from 5 to 15 min (Hsu and Bajaj, 2022), with oral corticosteroids beginning around 4 h (Rowe et al., 2001). The rapid and clinically meaningful improvement in lung function and dyspnea observed within 2–15 min in enrolled participants is promising. In studies where patients receive anti-muscarinic medication, these drugs may compete with VNS action by blocking acetylcholine from multiple sources beyond the vagal nerve (Liu et al., 2018). Compared to the physiological modulation exerted by VNS, anti-muscarinic agents provide a more complete blockade of acetylcholine action. The concomitant use of anti-muscarinic agents with VNS could represent either a theoretical limitation or a potential synergy, highlighting the need to examine this interaction further. Finally, the low number of non-severe AEs using low-voltage VNS supports the safety of the intervention in the setting of acute asthma exacerbations.

Further, although well-designed, the animal studies presented focus primarily on a guinea pig model, limiting translatability to human airway physiology. Guinea pig models have long been used to mimic the respiratory system in humans (McGovern and Mazzone, 2014). This animal model displays anatomical, physiological and pharmacological characteristics that closely resemble those of the human airway (McGovern and Mazzone, 2014). However, there are morphological differences between the guinea pig and human airway model that warrant caution in interpreting study results. Guinea pigs have fewer mucus glands than humans (McGovern and Mazzone, 2014). It is therefore not the best model to study the mucus secretion that is well established in human asthma pathophysiology. The abundant tachykinins in this animal model as well as the prominent role played by the central airways are also distinct features worth nothing (McGovern and Mazzone, 2014). Together, these data suggest that VNS for asthma should be further explored. Future studies should specifically focus on conducting a double-blind experimental RCT with a sham-controlled group and consistent anti-asthma treatment across intervention arms, where acute bronchoconstriction is triggered by a provocation challenge. This would allow confirmation of optimal stimulation parameters, duration of stimulation and timing to induce sustained benefits. Designing a double-blind RCT in patients with acute asthma exacerbation unresponsive to conventional management would also be of interest to document change from baseline differences in lung function parameters at distinct points in time, including after a prolonged observation period. Emerging closed-loop VNS paradigms may enable real-time adaptive modulation of vagal neural signals involved in systemic and respiratory control, while minimizing unwanted off-target parasympathetic or sympathetic effects (Lerman et al., 2025). This paradigm hold translational promise for studying therapeutic VNS in asthma, both for its bioneurological rationale and its potential future application in this respiratory condition.

## 5 Conclusion

This review summarizes the published animal and human data on the use of VNS in asthma. Based on limited, small, though well-designed animal trials, small pre-experimental human studies and one small randomized placebo-controlled study in adults with asthma, low-voltage VNS appears to have the potential to reduce acute bronchoconstriction. However, true contribution from VNS alone remains unknown given the concomitant use of anti-asthmatic therapies in published studies. Robust randomized placebo-controlled trials investigating the potential clinical utility of VNS in humans are warranted. Future study designs should incorporate objective asthma-related biomarkers (e.g., fractional exhaled nitric oxide (FeNO), blood and sputum eosinophils, methacholine challenge, and other respiratory or systemic inflammatory markers) to help isolate the specific effects of VNS and enhance comprehensive fundamental-to-clinical translatability.

## Data Availability

The original contributions presented in the study are included in the article/Supplementary Material, further inquiries can be directed to the corresponding author.

## Abbreviations

AE, adverse event; ED, emergency department; FEF, forced expiratory flow; FEV1, forced expiratory volume in 1 s; FVC, functional vital capacity; Hz, hertz; PEF, peak expiratory flow; iNANC, inhibitory non-adrenergic non-cholinergic; Ppi, pulmonary pressure inhalation; RCT, randomized controlled trial; VNS, vagus nerve stimulation; V, Volts; WOB, work of breathing.
