# Supplementary material for: Vagus nerve stimulation as a potential treatment for acute asthmatic bronchoconstriction: a systematic review
Source: Front Physiol. 2025 Aug 13;16:1625871. doi: 10.3389/fphys.2025.1625871 (PMC12380776; doi:10.3389/fphys.2025.1625871)
Supplement: Supplementary file 1 [file Table1.docx]

***Supplementary Material***

**Supplemental Table 1**. Search strategy

| Database | Search terms | Results |
| --- | --- | --- |
| PubMed | ("asthma"[MeSH] OR "cough"[MeSH] OR “respiratory system”[MeSH] OR “bronchoconstriction”[MeSH] OR “bronchial spasm”[MeSH] OR "asthma*"[tiab] OR "cough*"[tiab] OR “bronchoconstrict*”[tiab] OR “bronchial constrict*”[tiab] OR “bronchospasm*”[tiab] OR “bronchial spasm*”[tiab])  AND  ("vagus nerve stimulation"[MeSH] OR "deep brain stimulation"[MeSH] OR "vagus nerve stimulat*"[tiab] OR "vagal nerve stimulat*"[tiab] OR "deep brain stimulat*"[tiab] OR "neuromodulat*"[tiab] OR "neurostimulat*"[tiab] OR “VNS”[tiab] OR “DBS”[tiab]) | 637 |
| Embase | (‘asthma*’ OR ‘cough*’ OR ‘bronchoconstrict*’ OR ‘bronchial constrict*’ OR ‘bronchospasm*’ OR ‘bronchial spasm*’):ti,ab,kw  AND  (‘vagus nerve stimulat*’ OR ‘vagal nerve stimulat*’ OR ‘deep brain stimulat*’ OR ‘neuromodulat*’ OR ‘neurostimulat*’ OR ‘VNS’ OR ‘DBS’):ti,ab,kw | 484 |
| Scopus | TITLE-ABS-KEY( ("asthma*" OR "cough*" OR “bronchoconstrict*” OR “bronchial constrict*” OR “bronchospasm*” OR “bronchial spasm*”)  AND  ("vagus nerve stimulat*" OR "vagal nerve stimulat*" OR "deep brain stimulat*" OR "neuromodulat*" OR "neurostimulat*" OR “VNS” OR “DBS”)) | 951 |

| **Supplemental Table 2:** Critical appraisal of the included preclinical studies using the SYRCLE tool for animal studies. | | | | | | | | | | |
| --- | --- | --- | --- | --- | --- | --- | --- | --- | --- | --- |
| **Study** | **Type of bias** | | | | | | | | | |
|  | **Selection bias** | | | **Performance bias** | | **Detection bias** | | **Attrition bias** | **Reporting bias** | **Other** |
|  | 1. Sequence generation | 2. Baseline characteristics | 3. Allocation concealment | 4. Random housing | 5. Blinding | 6. Random outcome assessment | 7. Blinding | 8. Incomplete outcome data | 9. Selective outcome reporting | 10. Free of other sources of bias |
| Hoffmann et al. 2009 | N/A | Yes | N/A | Unclear | N/A | Unclear | Yes | Yes | Yes | Yes |
| Hoffman et al. 2012 | N/A | Yes | N/A | Unclear | N/A | Yes | Yes | Yes | Yes | Yes |
| *NA: not applicable for this study*   \| **Study** \| Baseline characteristics \| Patient’s History \| Presentation of clinical conditions \| Description of diagnostic tests and tools \| Description of treatment/intervention \| Description of post-intervention clinical condition \| Identification of adverse events \| Presentation of key takeaway lessons \| \| --- \| --- \| --- \| --- \| --- \| --- \| --- \| --- \| --- \| \| Sepulveda et al. 2008 \| Yes \| Yes \| Yes \| Yes \| Yes \| Yes \| Yes \| Yes \|   **Supplemental Table 3:** Critical appraisal of the included case report using the JBI tool for human studies. | | | | | | | | | | |

|  | |  | |  | | | | | | | |
| --- | --- | --- | --- | --- | --- | --- | --- | --- | --- | --- | --- |
| **Study** | **Type of bias** | | | | | | | | | | |
|  | **Study objectives** | | **Study design** | | | | **Study population** | **Intervention and co-intervention** | | | |
|  | Description of study aims, objectives, hypothesis | | Prospective study | | Multicenter study | Consecutive recruitment of patients | Description of participants’ characteristics | Description of eligibility criteria | Recruitment of patients at similar stages of the disease | Description of the intervention of interest | Description of co-interventions |
| Steyn et al. 2013 | Yes | | Yes | | Yes | Unclear | Partial | Yes | Unclear | Yes | Yes |
| Miner et al. 2012 | Yes | | Yes | | Yes | Yes | Yes | Yes | Yes | Yes | Yes |

**Supplemental Table 4:** Critical appraisal of the included case series using the IHE tool for human studi

| **Study** | **Type of bias** | | | | | | | | | | |
| --- | --- | --- | --- | --- | --- | --- | --- | --- | --- | --- | --- |
|  | **Outcome measures** | | | | **Statistical analysis** | **Results and conclusions** | | | | | **Competing interests and** |
|  | Establishment of relevant outcomes | Blinding of outcome assesors | Measurement of outcomes using appropriate methods | Outcome measures made before and after intervention | Use of appropriate statistical tools | Appropriate length of follow up | Reporting of participants lost to follow up | Estimates of random variability | Were adverse events reported | Conclusions supported by results | Competing interests and sources of support |
| Steyn et al. 2013 | Yes | No | Yes | Yes | No | Yes | Yes | No | Yes | Yes | Yes |
| Miner et al. 2012 | Yes | No | Yes | Yes | Yes | Yes | Yes | Yes | Yes | Yes | Yes |
